# Retraction Note to: Redox modification of cysteine residues regulates the cytokine activity of high mobility group box-1 (HMGB1)

**DOI:** 10.1186/s10020-020-00264-1

**Published:** 2020-12-30

**Authors:** Huan Yang, Peter Lundbäck, Lars Ottosson, Helena Erlandsson-Harris, Emilie Venereau, Marco E. Bianchi, Yousef Al-Abed, Ulf Andersson, Kevin J. Tracey, Daniel J. Antoine

**Affiliations:** 1 Laboratory of Biomedical Science, The Feinstein Institute for Medical Research, Manhasset, NY USA; 2 Departments of Women’s and Children’s Health, Medicine and Rheumatology Research Laboratory, Karolinska Institutet and Karolinska University Hospital, Stockholm, Sweden; 3 San Raffaele University and Scientific Institute, Milan, Italy; 4 Department of Medicinal Chemistry, The Feinstein Institute for Medical Research, Manhasset, NY USA; 5 Department of Molecular and Clinical Pharmacology, MRC Centre for Drug Safety Science, University of Liverpool, Liverpool, L69 3GE UK

## Retraction Note to: Mol Med (2012) 18:250–259 https://doi.org/10.2119/molmed.2011.00389

The Editors-in-Chief have retracted this article (Yang et al. 2012) following an investigation by the University of Liverpool. The investigation concluded that data contained in this paper demonstrate evidence of data manipulation and figure fabrication relating to mass-spectrometry data and are therefore unreliable. The investigation found no evidence that the mass-spectrometry was carried out on any University of Liverpool instrumentation from systematic examination of equipment logs. This does not exclude the fact that the mass-spectrometry may have been undertaken at another research center. However, they found no evidence that the mass-spectrometry was performed anywhere else or any financial audit trail to indicate that instrument time was paid for elsewhere. Figure 1 trace A1 demonstrates mismatches between the stated mass and where the spectra align and mislabeling of the y axis. In Fig. 1 trace A2, there are mismatches between the peaks and axis masses, inconsistencies in the presence of a zero in the second decimal place and the presence of hollow bars instead of lines for peaks. In Fig. 1 trace A3, there are mismatches between the peaks and the axis masses, inconsistencies in the graduation of the mz axis – there appears to have the graduation of another axis superimposed on it – and the presence of hollow bars instead of lines for peaks. In Fig. 1 trace A2 and A3, the traces have been modified, but there are duplicated areas still visible. In Fig. 1 trace B1, there are mismatches between the peaks and the axis masses, mislabeling of the y axis, inconsistencies in the graduation of the mz axis and the presence of hollow bars instead of lines for peaks. In Fig. 1 trace B2, Fig. 1 trace B3, Fig. 1 trace C1 and Fig. 1 trace C2, there are mismatches between the peaks and the axis masses and the presence of hollow bars instead of lines for peaks. The co-authors of the article were found by the investigation not to be complicit in any research misconduct, and they have been invited to resubmit a revised version of the manuscript for further peer review. More information on the university's investigation can be found on the university website (Further update on research misconduct investigation 2020).

All authors agree to this retraction.
